# Marked Disparities in Pre-Pregnancy Obesity and Overweight Prevalence among US Women by Race/Ethnicity, Nativity/Immigrant Status, and Sociodemographic Characteristics, 2012–2014

**DOI:** 10.1155/2019/2419263

**Published:** 2019-02-10

**Authors:** Gopal K. Singh, Jessica N. DiBari

**Affiliations:** ^1^US Department of Health and Human Services, Health Resources and Services Administration, Office of Health Equity, 5600 Fishers Lane, Room 13N42, Rockville, MD 20857, USA; ^2^US Department of Health and Human Services, Health Resources and Services Administration, Maternal and Child Health Bureau, Office of Epidemiology & Research, Division of Research, 5600 Fishers Lane, Room 18N120, Rockville, MD 20857, USA

## Abstract

This study examines racial/ethnic, nativity, and sociodemographic disparities in the prevalence of pre-pregnancy obesity and overweight in the United States. Logistic regression was fitted to the 2012–2014 national birth cohort data to derive unadjusted and adjusted differentials in pre-pregnancy obesity (BMI ≥30), severe obesity (BMI ≥40), and overweight/obesity (BMI ≥25) prevalence among 10.4 million US women of childbearing age. Substantial racial/ethnic differences existed, with pre-pregnancy obesity rates ranging from 2.6% for Chinese and 3.3% for Vietnamese women to 34.9% for American Indians/Alaska Natives (AIANs) and 60.2% for Samoans. Pre-pregnancy overweight/obese prevalence ranged from 13.6% for Chinese women to 61.7% for AIANs and 86.3% for Samoans. Compared to non-Hispanic whites, women in all Asian subgroups had markedly lower risks of pre-pregnancy obesity, severe obesity, and overweight/obesity, whereas Samoans, Hawaiians, AIANs, blacks, Mexicans, Puerto Ricans, and Central/South Americans had significantly higher risks. Immigrant women in each racial/ethnic group had lower rates of pre-pregnancy obesity than the US-born. Sociodemographic risk factors accounted for 33–47% of racial/ethnic disparities and 12–16% of ethnic-immigrant disparities in pre-pregnancy obesity and overweight/obesity. Further research is needed to assess the effects of diet, physical inactivity, and social environments in explaining the reported ethnic and nativity differences in pre-pregnancy obesity.

## 1. Introduction

Obesity rates have increased dramatically in the United States during the past four decades, with the prevalence having more than doubled for all racial/ethnic, gender, immigrant, and socioeconomic groups [1–4]. Currently, 38% of US men and 40% of US women are classified as obese (body mass index (BMI) ≥30 kg/m^2^), and more than 70% of the US adult population are considered overweight or obese (BMI ≥25 kg/m^2^) [1, 2]. Rates of obesity and overweight/obesity for the US adult population are projected to reach 43% and 77%, respectively, by 2020 [4].

According to the national data, obesity prevalence among US women of childbearing age (18–49) increased nearly 4-fold, from 7.4% in 1976 to 27.5% in 2014; the overweight/obese prevalence rose from 22.8% in 1976 to 53.5% in 2014 [3, 5]. Nearly 4 million women give births each year in the US, and rates of pre-pregnancy obesity among mothers are high with a prevalence >20% [6, 7]. Pre-pregnancy obesity is associated with increased risk for a number of adverse pregnancy and birth outcomes, including gestational diabetes, preeclampsia, gestational hypertension, cesarean section, dysfunctional and prolonged labor, induced labor, miscarriage, stillbirth, fetal macrosomia, preterm birth, select birth defects, and infant mortality [7–14]. Women with pre-pregnancy obesity are also at greater lifetime risks for developing chronic hypertension and type 2 diabetes than those of normal weight [11]. Children born to women with pre-pregnancy obesity are at increased risk of obesity during childhood and adult life [9, 11, 13].

Data from the Pregnancy Risk Assessment and Monitoring System (PRAMS) indicate a significant rise in the prevalence of pregnancy-related obesity in 20 US states, increasing from 17.6% in 2003 to 20.5% in 2009 [7]. Studies using PRAMS and birth certificate data show highest rates of pre-pregnancy obesity among non-Hispanic black women and American Indians/Alaska Native (AIAN) women, followed by Hispanics, non-Hispanic whites, and Asian/Pacific Islanders (APIs) [7, 13, 15, 16]. Socioeconomic differences in pre-pregnancy obesity have also been reported, with women at lower education and income levels having markedly higher prevalence [13, 16].

Despite the many adverse health effects of obesity in the preconception period, detailed racial/ethnic and sociodemographic risk factors associated with increased risk of pre-pregnancy obesity are not well documented in the US. While maternal pre-pregnancy obesity prevalence for broad racial/ethnic groups has previously been reported [7, 13, 15–17], disparities in pre-pregnancy obesity and overweight among specific API, Hispanic, and immigrant subgroups are not analyzed. Moreover, although characteristics such as maternal age, parity, marital status, education, and place of residence have been mentioned as possible risk factors for maternal obesity, few studies have examined racial/ethnic and immigrant disparities adjusted for these factors [7, 12, 13]. A better understanding of pre-pregnancy obesity risks and their determinants among major racial/ethnic and immigrant groups is vital to improving preconception health and health outcomes among mothers and children. Targeted and culturally relevant interventions for obesity prevention and control can reduce overall health disparities, improve intergenerational weight trends, and promote health equity for the nation [3, 4, 8].

The primary aim of this study is to examine the extent of racial/ethnic and immigrant disparities in the prevalence of pre-pregnancy obesity and overweight among US women and to identify relevant sociodemographic risk factors, using the latest national birth cohort data. The study also explores a wider range of social inequalities by examining whether racial/ethnic disparities in pre-pregnancy obesity and overweight vary according to nativity/immigrant status and maternal education. Since immigration is a major characteristic of the Asian and Hispanic populations and nearly a quarter of all US births occur among foreign-born mothers [6], our analysis is also stratified by nativity status to highlight immigrant differences in obesity rates within each racial/ethnic group.

## 2. Methods

Maternal pre-pregnancy BMI data in this study are derived from the birth certificates filed in the 50 states, New York City, and the District of Columbia (DC) [6, 18]. These data have been included in the annual natality files by the National Center for Health Statistics, Centers for Disease Control and Prevention (CDC), for selected states since 2003. The number of states reporting pre-pregnancy BMI data increased from 40 in 2012 to 42 in 2013 and 48 in 2014 [6, 18]. The annual natality file includes birth certificate data for about 4 million births that occur in the United States each year [6, 18]. The *US Standard Certificate of Live Birth* is the basis for the national birth data [6].

In addition to pre-pregnancy BMI and detailed race/ethnicity, the birth certificate data include maternal and paternal age, nativity/immigrant status, marital status, education, place of residence, parity/birth order, birth interval, birthweight, gestational age, congenital anomalies of the newborn, tobacco and alcohol use during pregnancy, prenatal care utilization, gestational weight gain, method of delivery (vaginal or cesarean), attendant at birth, pregnancy history, and a variety of medical risk factors and complications such as pre-pregnancy and gestational diabetes, chronic and pregnancy-induced hypertension, eclampsia, uterine bleeding, placenta previa, prolonged labor, and induction of labor. Detailed descriptions of the birth certificate data and national natality files are available elsewhere [1, 6, 18, 19].

We used the 2012–2014 national birth cohort data [6, 18]. During 2012–2014, 11,873,098 births occurred among US mothers. Approximately 12.1% of the births did not include pre-pregnancy maternal BMI data, which included several states that did not report BMI data during the three years [18]. The sample for the analysis included 10,431,092 live births. Of these, 2,529,920 births occurred among mothers with pre-pregnancy obesity, 5,191,191 births among mothers with pre-pregnancy overweight/obesity, and 459,283 births among mothers with severe obesity. Aggregating data for three years ensured sufficient sample sizes for analyzing obesity patterns among groups stratified by race/ethnicity, immigrant status, and maternal education. Consistent with the WHO and CDC guidelines, pre-pregnancy overweight/obesity was defined as a BMI ≥25 kg/m^2^, obesity as a BMI ≥30 kg/m^2^, and severe or grade 3 obesity as a BMI ≥40 kg/m^2^ [1–4, 20]. Note that the overweight/obesity category includes obese women.

Race/ethnicity was classified into 17 major categories: Non-Hispanic whites, Non-Hispanic blacks, AIANs, Chinese, Asian Indians, Filipinos, Japanese, Koreans, Vietnamese, Hawaiians, Samoans, and other Asian/Pacific Islanders, Mexicans, Puerto Ricans, Cubans, Central and South Americans, and other Hispanics. Immigrant status was defined on the basis of mothers' place of birth [6, 18, 19]. US-born were those born in one of the 50 states or Washington, DC. Immigrants or foreign-born refer to those born outside these territories [6, 18, 19]. The joint variable of ethnic-immigrant status included 31 categories, with each racial/ethnic group divided into the US-born and foreign-born categories. Note that although AIANs, Hawaiians, and Samoans are considered native-born in the present analysis, a small percentage of AIANs and Hawaiians and 33% of Samoans are born outside the 50 states and Washington, DC [6, 19].

In addition to race/ethnicity and immigrant status, we considered the following sociodemographic factors that have been associated with obesity risk and were available in the natality files: maternal age, parity, marital status, metropolitan/nonmetropolitan residence, geographic region of residence, and maternal education [3, 4, 12, 13, 15, 16]. These covariates were measured as shown in Tables 1 and 2.

Multivariate logistic regression was used to model the adjusted association between each sociodemographic characteristic and the risk of pre-pregnancy obesity and overweight [21]. In estimating odds of obesity or overweight for race/ethnicity and ethnic-immigrant status, we considered white women or US-born white women as the reference group based on prior research and because they represent the majority population [1, 19].

We used root-mean-square deviation (RMSD) as a summary measure of health disparities among 17 racial/ethnic and 31 ethnic-immigrant groups [4]. The RMSD is similar to the square root of the variance, except that the average-squared deviations are calculated using a “standard” estimate other than the sample mean. The RMSD is given by the formula:

(1) $$\begin{array}{c} \text{RMSD}=\text{SQRT}\left\{\sum_{i}\frac{{\left({\text{O}}_{\text{ri}}-{\text{O}}_{\text{rl}}\right)}^{2}}{\text{I}}\right\}, \end{array}$$

where O_ri_ is the obesity or overweight/obesity prevalence for the *i*th group (*i*=1,2, and, 31), O_rl_ is the corresponding statistic for the “standard” group (total US population) or group with the lowest prevalence (i.e., Chinese women), and I is the number of racial/ethnic (17) or ethnic-immigrant groups (31) being compared.

While RMSD is a measure of absolute health disparity, the coefficient of variation (CV) of the RMSD provides an estimate of relative disparity and is given by

(2) $$\begin{array}{c} \text{CV}\left(\text{RMSD}\right)=\left(\frac{\text{RMSD}}{{\text{O}}_{\text{rl}}}\right)\times 100,\;{\text{O}}_{\text{rl}}>0. \end{array}$$

In both the obesity and overweight/obesity models, we examined interactions of race/ethnicity with nativity and maternal education by estimating logistic models that included the joint variables of ethnicity-immigrant status and race/ethnicity-education. Fitted logistic models were used to derive adjusted obesity and overweight prevalence at mean values of the covariates [4, 21].

## 3. Results

### 3.1. Racial/Ethnic Disparities in Pre-Pregnancy Obesity and Overweight

During 2012–2014, the overall prevalence of pre-pregnancy obesity and overweight/obesity among US mothers was 24.3% and 49.8%, respectively. Of all racial/ethnic groups, Samoan women had the highest prevalence of pre-pregnancy obesity (60.2%), followed by AIANs (34.9%), blacks (33.9%), Hawaiians (32.4%), Puerto Ricans (28.6%), and Mexicans (28.0%) (Table 1). Chinese women had the lowest pre-pregnancy obesity prevalence at 2.6%. Indeed, all Asian subgroups, including Chinese, Japanese, Vietnamese, Koreans, Asian Indians, and Filipinos, had markedly lower pre-pregnancy obesity prevalence than non-Hispanic whites (22.3%). Mexicans and Puerto Ricans had significantly higher prevalence of pre-pregnancy obesity than non-Hispanic white women.

**Figure 1:**
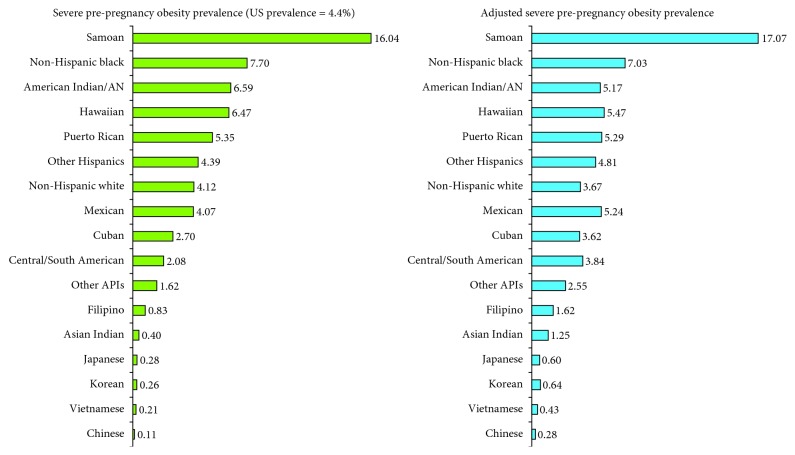
Racial/ethnic disparities in observed and adjusted^1^ prevalence (%) of severe pre-pregnancy obesity (BMI ≥40) among US women, 2012–2014. ^1^Adjusted for maternal age, parity, marital status, nativity/immigrant status, maternal education, and place and region of residence.

Pre-pregnancy overweight/obesity prevalence ranged from a low of 13.6% for Chinese women to 61.7% for AIANs and 86.3% for Samoans (Table 2). Overweight/obesity prevalence exceeded 55% for Puerto Ricans, Mexicans, Hawaiians, and blacks but was lower than 20% for Vietnamese, Japanese, and Korean mothers. The overall prevalence of severe pre-pregnancy obesity among US mothers was 4.4% (Figure 1). Chinese women had the lowest prevalence of severe obesity (0.1%), with all major Asian subgroups reporting a prevalence of <1%. Samoans had the highest prevalence of severe obesity (16.0%), followed by blacks (7.7%), AIANs (6.6%), Hawaiians (6.5%), and Puerto Ricans (5.4%).

Racial/ethnic groups varied greatly in their sociodemographic characteristics that are associated with pre-pregnancy obesity (Table 3). For example, while <12% of births occurred among AIAN, Puerto Rican, and black mothers aged ≥35 years, this percentage was 35% among Chinese and 49% among Japanese mothers. More than 19% of Hawaiian, Samoan, and AIAN mothers reported previously having 4 or more births, compared to <3% of Chinese and Asian Indian mothers. Educational attainment was highest among Asian Indian and Korean women and lowest among Mexican and Samoan women. The percentage of mothers with a college degree ranged from 75.3% for Koreans and 77.0% for Asian Indians to 7.5% for Samoans, 8.4% for Mexicans, and 9.3% for AIANs. More than 87% of Chinese and Asian Indian mothers were foreign-born, compared with 6.2% of non-Hispanic whites and 14.2% of blacks. The proportion of births outside marriage was much higher among blacks, AIANs, and Hispanics compared to white and Asian women. The percentage of unmarried mothers ranged from 3.5% for Asian Indians and 7.0% for Japanese to 63.4% for Puerto Ricans, 66.3% for AIANs, and 71.4% for blacks. More than 55% of Hawaiian, Japanese, Filipino, and Samoan mothers resided in the Western region, compared to 7.7% of Puerto Ricans and 8.7% of blacks.

After controlling for sociodemographic factors, Central/South Americans, Puerto Ricans, Mexicans, Hawaiians, AIANs, and non-Hispanic blacks had 26–82% higher odds of pre-pregnancy obesity and 48–86% higher odds of overweight/obesity compared to non-Hispanic white women (Tables 1 and 2). Samoans had 6.1 times higher adjusted odds of pre-pregnancy obesity and 8.0 times higher adjusted odds of overweight/obesity compared to non-Hispanic whites. Blacks and Samoans, respectively, had 2.0 and 5.7 times higher adjusted odds of severe obesity than non-Hispanic whites (data not shown).

Non-Hispanic white women, on the other hand, had 5.8 higher adjusted odds of pre-pregnancy obesity, 3.7 times higher adjusted odds of overweight/obesity, and 14.3 times higher adjusted odds of severe obesity compared to Chinese women. Indeed, compared to non-Hispanic whites, women in all Asian subgroups had 21–93% lower odds of pre-pregnancy obesity, severe obesity, and overweight/obesity.

Tables 1 and 2 show variation in prevalence and odds of pre-pregnancy obesity and overweight/obesity according to other sociodemographic characteristics. Increasing maternal age and parity, lower education, US-born status, nonmetropolitan residence, and residence in the Midwest and Southern regions were independently associated with pre-pregnancy obesity and overweight. Mothers aged 40–44 years had 3.5 times higher adjusted odds of pre-pregnancy obesity and 3.2 times higher adjusted odds of overweight/obesity than those aged <20 years. US-born mothers had 86% higher adjusted odds of pre-pregnancy obesity and 35% higher odds of overweight/obesity than immigrant mothers. Mothers in the Midwest and Southeast regions had 22–34% higher adjusted odds of pre-pregnancy obesity and overweight/obesity than those in the Mountain region. Mothers with lower education levels had two times higher adjusted odds of pre-pregnancy obesity than those with a college degree. Risk of pre-pregnancy obesity and overweight/obesity increased consistently with increasing maternal parity. Based on disparity indices, sociodemographic factors accounted for 33%, 47%, and 61% of the racial/ethnic disparities in pre-pregnancy overweight/obesity, obesity, and severe obesity, respectively.

### 3.2. Ethnic-Immigrant Disparities in Pre-Pregnancy Obesity and Overweight/Obesity

**Figure 2:**
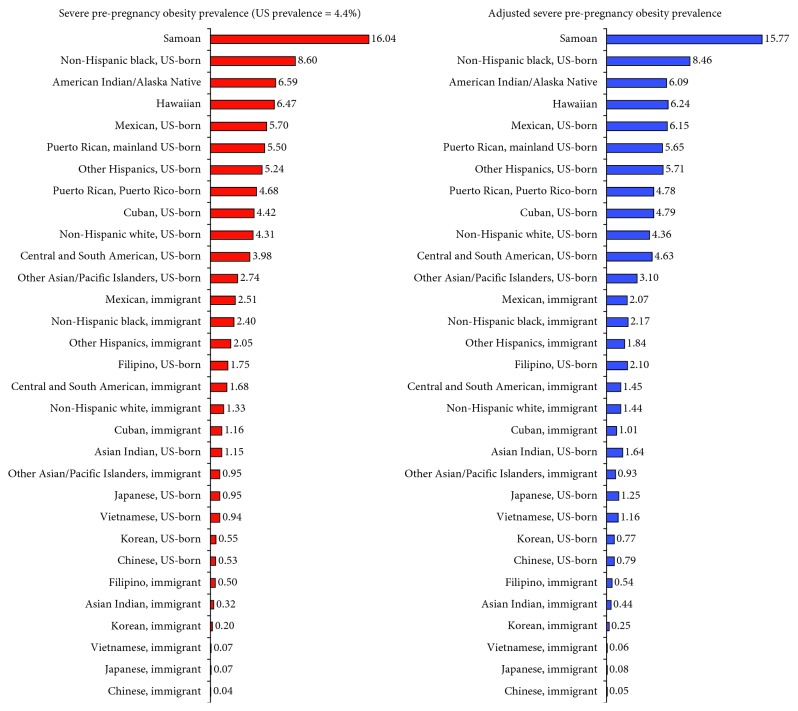
Ethnic-immigrant disparities in observed and adjusted^1^ prevalence (%) of severe pre-pregnancy obesity (BMI ≥40) among US women, 2012–2014. ^1^Adjusted for maternal age, parity, marital status, maternal education, and place and region of residence.

Pre-pregnancy obesity ranged from 1.9% for Chinese immigrants to 35.7% for US-born blacks and 60.2% for Samoans (Table 4). US-born Chinese women had 3.7 times higher risk of obesity than Chinese immigrants, while US-born black women had 51% higher risk of obesity than immigrant black women. Overweight/obesity prevalence ranged from 10.5% for Japanese immigrants and 12.1% for Chinese immigrants to 61.7% for US-born blacks and AIANs and 86.3% for Samoans (Table 5). US-born Chinese and Japanese women had 2 and 3 times higher risks of overweight/obesity compared to their foreign-born counterparts, respectively. Prevalence of severe pre-pregnancy obesity varied from a low of <0.50% for Chinese, Japanese, Vietnamese, Korean, Asian Indian, and Filipino immigrants to a high of 8.6% for US-born blacks and 16.0% for Samoans (Figure 2).

Ethnic-immigrant disparities in obesity risks were greater than disparities shown by race/ethnicity alone. Compared with US-born whites, the adjusted odds of obesity were 49% lower for white immigrants, 83% higher for US-born blacks, 6% lower for black immigrants, 61% higher for US-born Mexicans, and 8% lower for Mexican immigrants (Table 4). Compared with US-born whites, all US-born and foreign-born Asian subgroups had 19–93% lower adjusted odds of obesity. Compared with US-born whites, the adjusted odds of overweight/obesity were 36% lower for white immigrants, 83% lower for Chinese immigrants, and 57% lower for US-born Chinese (Table 5). All Asian nativity groups had significantly lower adjusted risks of overweight/obesity than US-born whites. Compared with US-born whites, the adjusted odds of severe obesity were 4.2 times higher for Samoans, 2.1 times higher for US-born blacks, and 1.2 times higher for AIANs. However, compared with US-born whites, the adjusted odds of severe obesity was 68% lower for white immigrants and 52% lower for black immigrants. All Asian nativity groups had considerably lower adjusted risks of severe obesity than US-born whites (data not shown). Sociodemographic factors accounted for 12%, 16%, and 21% of ethnic-immigrant disparities in pre-pregnancy overweight/obesity, obesity, and severe obesity, respectively.

### 3.3. Ethnic-Specific Educational Gradients in Pre-Pregnancy Obesity and Overweight

Marked educational inequalities in pre-pregnancy obesity and overweight/obesity were generally found for all broad racial/ethnic groups, with mothers with less than a college education having significantly higher obesity and overweight/obesity prevalence than their counterparts with a college degree (Table 6). Obesity prevalence ranged from 6.2% for API women with a college degree to over 37% for black and AIAN women with 13–15 years of education. Overweight/obesity prevalence ranged from 25.3% for API women with a college degree to over 64% for black and AIAN women with 13–15 years of education. Compared with API women with a college degree, the adjusted odds of obesity were almost 4 times higher for white women with less than a college degree and 5–7 times higher for Hispanic, AIAN, and black women with less than a college degree. The patterns in overweight/obesity risks were similar. Within each educational stratum, significant racial/ethnic disparities in obesity and overweight risks existed.

## 4. Discussion

To our knowledge, this is the largest population-based study of pre-pregnancy obesity in the US. We have shown marked disparities in pre-pregnancy obesity among a large number of racial/ethnic and immigrant groups in the US, maternal obesity risks for many of which were not previously examined. For several racial/ethnic groups, pre-pregnancy obesity levels were found to be very high, such as Hawaiians, blacks, AIANs, and Samoans who have a prevalence of 32%, 34%, 35%, and 60%, respectively. Indeed, more than 86% of Samoan women were classified as overweight or obese entering into pregnancy, with the prevalence exceeding 60% for Hawaiians, AIANs, and US-born black women. The results of our national study indicate substantial racial/ethnic and nativity disparities in the risk of maternal pre-pregnancy obesity and overweight, which were only partially explained by socioeconomic and demographic differences.

The increased risk of pre-pregnancy obesity observed here for several ethnic-minority and socially disadvantaged groups such as AIANs, blacks, Mexicans, Puerto Ricans, Hawaiians, and Samoans is consistent with prior studies reporting high levels of obesity and overweight among adult women in these subgroups [3, 4, 7, 13, 16, 19]. Similarly, low levels of obesity and overweight among specific Asian subgroups such as Chinese, Japanese, Vietnamese, Koreans, Asian Indians, and Filipinos are consistent with those reported in previous studies on adult obesity in the US [3, 4, 19]. Previous studies have shown Hispanics to be at an increased risk of pre-pregnancy obesity and adult obesity [1–3, 7, 13, 16]. Compatible with adult obesity patterns, our analysis showed particularly high risks of pre-pregnancy obesity among Mexicans and Puerto Ricans compared to non-Hispanic whites and Asian subgroups [3, 18].

Substantial geographic variation in adult obesity prevalence has previously been reported, and our findings of higher prevalence of pre-pregnancy obesity in the Midwest and Southern regions are consistent with the previously reported regional patterns [1, 3, 22]. Increased risk of pre-pregnancy obesity associated with lower maternal education is consistent with studies showing association between low socioeconomic status and higher obesity levels in women [1, 3, 13, 16, 22]. Diet, physical activity, genetic, and social environmental factors could account for ethnic, immigrant, and socioeconomic disparities in pre-pregnancy obesity [3, 4, 7, 8, 11, 23].

Although immigrants account for 13% of the total US population, immigrant women make up approximately 20% of the reproductive-age population [24]. Given the profound inequalities in obesity by nativity/immigrant status shown here and in previous studies and the fact that immigrants represent a larger percentage of the population aged 15–49 than all ages, the magnitude of health disparities is likely to be greater for women in reproductive ages than for the general population, all else being equal [3, 4, 19]. Although immigrant women in each racial/ethnic group had lower rates of pre-pregnancy obesity and overweight than the US-born, their reduced obesity risks and other health advantages are likely to diminish with increasing acculturation levels or duration of residence in the US [3, 4, 19, 25]. Although genetic factors might partly explain racial/ethnic disparities in pre-pregnancy obesity, findings from previous studies on migrant health as well as lower obesity risks among immigrants of similar ethnicities seen here might indicate the significance of social environments, acculturation, and lifestyle factors [3, 4, 19, 25]. Ethnic-minority and socially disadvantaged groups in the US differ greatly from the majority, affluent groups in their social, physical, and living environments. They have limited access to neighborhood amenities such as sidewalks, parks/playgrounds, green spaces, public transportation, and healthy, affordable foods that promote physical activity, healthy lifestyle, and healthy living [3, 4, 25, 26].

Our study has some limitations. Because of lack of data, important risk factors for obesity such as diet, physical activity, and the social and built environments could not be taken into account. Moreover, prevalence estimates of pre-pregnancy obesity and overweight were for women who had a live birth during 2012–2014 and excluded women who became pregnant but experienced fetal loss, miscarriages, or abortions [6, 18]. Since pre-pregnancy obesity in women is associated with these adverse perinatal outcomes, the reported pre-pregnancy obesity prevalence is likely to be underestimated [7]. Additionally, since pre-pregnancy weight on the birth certificate is self-reported by the mothers, pre-pregnancy obesity and overweight prevalence is likely to be underestimated [7]. The advantages of our study are its large sample size, national representativeness, and the ability to estimate pre-pregnancy obesity prevalence for a large number of racial/ethnic and immigrant groups, allowing for a more nuanced understanding of ethnic and socioeconomic disparities.

### 4.1. Implications for Practice and Policy

Factors such as inequalities in the social and built environments, physical activity levels, and dietary behavior are likely determinants of the racial/ethnic and nativity disparities in maternal pre-pregnancy obesity shown here [3, 4, 7, 11, 23, 26, 27]. Regular obstetric/gynecologic encounters offer an opportunity for behavioral counseling and lifestyle interventions to improve dietary intake and physical activity from preconception through pregnancy and well into the postpartum period [7, 11, 13, 28, 29]. Clinical guidelines are necessary to make improvements to the preconception, pregnancy, and postpartum visits [30, 31]. Interventions during these frequent medical encounters may be an effective strategy to counteract the rising rates of maternal obesity in the US [11, 29, 30]. Obesity is a leading health-risk factor for the nation and is associated with excess morbidity and mortality [2, 3, 32]. Due to high prevalence, a rapidly increasing trend, and large social-group disparities, obesity is a major public health concern due to its linkage to a myriad of chronic health conditions and the considerable toll it takes on healthcare costs [2, 3, 32, 33]. Marked social inequalities in pre-pregnancy obesity among US women contribute to persistent disparities in maternal and child health and overall health.

The recent release of the *US Dietary Guidelines for Americans 2015–2020* provides evidence-based recommendations for health and nutrition. Counterpart guidelines for women of reproductive age are necessary to provide specific recommendations for this subpopulation [34]. The nutritional needs for women vary during pregnancy to provide adequate nutrients to the fetus and postpartum particularly among lactating women. In addition, obese women tend to give rise to infants who are of large-for-gestational age [35]. Healthy eating patterns and regular physical activity can help women, pre-pregnancy, during pregnancy, and postpartum, to optimize fetal growth and development, thus changing the intergenerational trajectory of obesity.

## 5. Conclusions

This large population-based study of 10.4 million US women has shown considerable heterogeneity in pre-pregnancy obesity and overweight risks across various racial/ethnic and immigrant groups. Some of the ethnic-minority groups such as Samoans, Hawaiians, Puerto Ricans, Mexicans, AIANs, and non-Hispanic blacks have relatively high levels of maternal obesity, ranging from 32% to 60%. High rates of pre-pregnancy obesity correspond closely with the increased risks of various adverse maternal and perinatal outcomes among these groups such as gestational hypertension and diabetes, preeclampsia, pregnancy complications, preterm birth, and infant mortality. Immigrants have substantially lower rates of pre-pregnancy obesity and overweight than their US-born counterparts regardless of race/ethnicity. These findings highlight the significance of stratifying obesity analyses by immigrant status and suggest ethnic group-specific and culturally appropriate interventions to prevent obesity in women of reproductive age and to improve health outcomes [3, 4, 19, 23]. Further research is needed to assess the role of sociobehavioral and environmental factors responsible for ethnic, immigrant, and sociodemographic disparities in maternal pre-pregnancy obesity.

## Figures and Tables

**Table 1:** Observed prevalence and logistic regressions showing unadjusted and covariate-adjusted differentials in pre-pregnancy obesity (BMI ≥30) among reproductive-age women in major racial/ethnic groups and by selected sociodemographic characteristics, United States, 2012–2014 (*N*=10, 431, 092)

Covariate	Number of births	Prevalence percent	Prevalence ratio	Model 1^1^			Model 2^2^			Covariate-adjusted	
				OR	95% CI		OR	95% CI		Prevalence	SE
*Race/ethnicity*											
Non-Hispanic white	57,28,722	22.31	1.00	1.00	Reference		1.00	Reference		21.09	0.02
Non-Hispanic black	15,23,283	33.94	1.52^*∗*^	1.79	1.79	1.79	1.82	1.82	1.82	32.25	0.04
American Indian/AN	96,037	34.86	1.56^*∗*^	1.86	1.86	1.87	1.65	1.64	1.65	30.19	0.14
Chinese	1,44,479	2.58	0.12^*∗*^	0.09	0.09	0.10	0.17	0.17	0.18	4.57	0.07
Japanese	18,074	4.25	0.19^*∗*^	0.15	0.15	0.16	0.25	0.24	0.27	6.54	0.22
Filipino	83,470	10.94	0.49^*∗*^	0.43	0.43	0.43	0.68	0.67	0.68	15.50	0.14
Hawaiian	2,041	32.44	1.45^*∗*^	1.67	1.57	1.79	1.58	1.48	1.69	29.32	0.96
Asian Indian	1,63,921	9.25	0.41^*∗*^	0.36	0.35	0.36	0.76	0.76	0.76	16.99	0.12
Korean	42,101	4.80	0.22^*∗*^	0.18	0.17	0.18	0.32	0.32	0.33	8.17	0.17
Vietnamese	59,579	3.26	0.15^*∗*^	0.12	0.11	0.12	0.18	0.17	0.18	4.68	0.10
Samoan	5,336	60.23	2.70^*∗*^	5.28	5.11	5.44	6.10	5.91	6.30	60.08	0.66
Other API	1,55,158	15.64	0.70^*∗*^	0.65	0.64	0.65	0.90	0.89	0.90	19.37	0.11
Mexican	14,91,966	27.95	1.25^*∗*^	1.35	1.35	1.35	1.66	1.66	1.66	30.29	0.04
Puerto Rican	1,61,294	28.60	1.28^*∗*^	1.40	1.39	1.40	1.52	1.51	1.52	28.51	0.11
Cuban	51,202	18.98	0.85^*∗*^	0.82	0.81	0.82	1.05	1.05	1.06	21.95	0.19
Central/South American	3,17,066	19.92	0.89^*∗*^	0.87	0.87	0.87	1.26	1.26	1.26	25.04	0.09
Other Hispanics	3,87,363	27.04	1.21^*∗*^	1.29	1.29	1.29	1.48	1.48	1.48	28.09	0.07

*Maternal age (years)*											
<20	7,35,009	16.06	1.00	1.00	Reference		1.00	Reference		12.47	0.04
20–24	23,75,337	24.74	1.54^*∗*^	1.72	1.71	1.73	1.84	1.83	1.86	20.42	0.03
25–29	29,91,749	25.54	1.59^*∗*^	1.79	1.78	1.81	2.51	2.49	2.53	25.58	0.03
30–34	27,47,472	23.89	1.49^*∗*^	1.64	1.63	1.65	2.81	2.79	2.83	27.66	0.03
35–39	12,75,698	25.31	1.58^*∗*^	1.77	1.76	1.78	3.22	3.20	3.25	30.31	0.05
40–44	2,84,881	26.63	1.66^*∗*^	1.90	1.88	1.92	3.47	3.43	3.51	31.77	0.09
≥45	20,946	23.34	1.45^*∗*^	1.59	1.54	1.64	3.15	3.04	3.26	29.84	0.33

*Parity*											
0	41,06,378	20.18	1.00	1.00	Reference		1.00	Reference		22.72	0.02
1	33,02,759	24.38	1.21^*∗*^	1.27	1.27	1.28	1.12	1.12	1.13	24.72	0.02
2	17,36,143	27.81	1.38^*∗*^	1.52	1.51	1.53	1.15	1.14	1.15	25.10	0.03
3	7,36,876	31.06	1.54^*∗*^	1.78	1.77	1.79	1.20	1.19	1.21	25.92	0.05
≥4	5,07,957	34.17	1.69^*∗*^	2.05	2.03	2.06	1.23	1.22	1.24	26.41	0.06

*Marital status*											
Married	62,25,913	22.29	1.00	1.00	Reference		1.00	Reference		24.03	0.02
Unmarried	42,05,179	27.16	1.22^*∗*^	1.30	1.30	1.30	1.03	1.03	1.03	24.54	0.02

*Nativity/immigrant status*											
US-born	81,19,717	26.10	1.47^*∗*^	1.64	1.64	1.65	1.86	1.85	1.87	26.45	0.02
Foreign-born	22,80,862	17.71	1.00	1.00	Reference		1.00	Reference		16.62	0.03

*Maternal education (years)*											
<12	16,33,121	25.53	1.60^*∗*^	1.80	1.80	1.81	2.02	2.01	2.03	27.55	0.04
12	25,69,666	28.18	1.76^*∗*^	2.07	2.06	2.07	2.09	2.08	2.10	28.23	0.03
13–15	30,44,340	28.73	1.80^*∗*^	2.12	2.11	2.13	1.99	1.98	2.00	27.25	0.03
≥16	30,79,059	15.97	1.00	1.00	Reference		1.00	Reference		16.17	0.02

*Place of residence*											
Metropolitan county	81,60,502	23.16	1.00	1.00	Reference		1.00	Reference		23.33	0.02
Non-metropolitan county	22,70,590	28.19	1.22^*∗*^	1.30	1.30	1.31	1.26	1.25	1.26	27.43	0.03

*Region of residence*											
New England	2,71,499	20.44	0.94^*∗*^	0.93	0.92	0.94	1.16	1.15	1.18	23.81	0.08
Mid-Atlantic	10,98,599	21.61	0.99^*∗*^	0.99	0.99	1.00	1.15	1.14	1.15	23.53	0.04
East Northcentral	16,27,751	26.33	1.21^*∗*^	1.29	1.28	1.30	1.34	1.33	1.35	26.25	0.03
West Northcentral	8,04,150	25.21	1.16^*∗*^	1.22	1.21	1.22	1.26	1.25	1.27	25.15	0.05
South Atlantic	19,61,847	24.84	1.14^*∗*^	1.19	1.18	1.20	1.19	1.18	1.20	24.19	0.03
East Southcentral	5,38,839	27.43	1.26^*∗*^	1.36	1.35	1.37	1.28	1.27	1.29	25.44	0.06
West Southcentral	15,43,133	25.24	1.16^*∗*^	1.22	1.21	1.23	1.16	1.15	1.17	23.74	0.03
Mountain	7,35,534	21.72	1.00	1.00	Reference		1.00	Reference		21.29	0.05
Pacific	18,49,740	22.78	1.05^*∗*^	1.06	1.06	1.07	1.17	1.16	1.18	23.89	0.03

OR = odds ratio; CI = confidence interval; AN = Alaska Native; API = Asian/Pacific Islander. ^*∗*^Statistically significant at *p* < 0.05. ^1^Unadjusted for the effects of other covariates. ^2^Adjusted for race/ethnicity, maternal age, parity, marital status, nativity, maternal education, and place and region of residence. Source: data derived from the 2012–2014 US National Natality data files.

**Table 2:** Observed prevalence and logistic regressions showing unadjusted and covariate-adjusted differentials in pre-pregnancy overweight or obesity (BMI ≥25) among reproductive-age women in major racial/ethnic groups and by selected sociodemographic characteristics, United States, 2012–2014 (*N*=10, 431, 092).

Covariate	Prevalence percent	Prevalence ratio	Model 1^1^			Model 2^2^			Covariate-adjusted	
			OR	95% CI		OR	95% CI		Prevalence	SE
*Race/ethnicity*										
Non-Hispanic white	46.37	1.00	1.00	Reference		1.00	Reference		45.15	0.02
Non-Hispanic black	60.88	1.31^*∗*^	1.80	1.80	1.80	1.86	1.86	1.86	59.92	0.04
American Indian/Alaska Native	61.69	1.33^*∗*^	1.86	1.85	1.87	1.73	1.72	1.73	58.22	0.16
Chinese	13.63	0.29^*∗*^	0.18	0.18	0.19	0.27	0.26	0.27	18.57	0.12
Japanese	15.20	0.33^*∗*^	0.21	0.20	0.21	0.27	0.27	0.28	18.91	0.31
Filipino	34.27	0.74^*∗*^	0.60	0.60	0.61	0.79	0.79	0.80	39.66	0.17
Hawaiian	61.29	1.32^*∗*^	1.83	1.70	1.97	1.76	1.63	1.90	58.72	1.08
Asian Indian	35.94	0.78^*∗*^	0.65	0.65	0.65	1.07	1.07	1.08	46.82	0.13
Korean	19.26	0.42^*∗*^	0.28	0.27	0.28	0.40	0.40	0.41	25.40	0.23
Vietnamese	14.84	0.32^*∗*^	0.20	0.20	0.20	0.25	0.25	0.25	17.57	0.17
Samoan	86.28	1.86^*∗*^	7.27	6.82	7.75	7.95	7.45	8.48	86.02	0.48
Other Asian/Pacific Islander	40.38	0.87^*∗*^	0.78	0.78	0.79	0.95	0.95	0.96	43.97	0.13
Mexican	57.95	1.25^*∗*^	1.59	1.59	1.59	1.81	1.81	1.81	59.36	0.05
Puerto Rican	55.41	1.19^*∗*^	1.44	1.43	1.44	1.56	1.56	1.56	55.82	0.12
Cuban	46.44	1.00^*∗*^	1.00	0.99	1.01	1.19	1.18	1.20	49.37	0.22
Central and South American	50.93	1.10^*∗*^	1.20	1.20	1.20	1.48	1.48	1.48	54.53	0.09
Other Hispanic	55.18	1.19^*∗*^	1.42	1.42	1.43	1.61	1.61	1.61	56.55	0.08

*Maternal age (years)*										
<20	38.47	1.00	1.00	Reference		1.00	Reference		32.78	0.06
20–24	49.54	1.29^*∗*^	1.57	1.56	1.58	1.67	1.66	1.67	44.11	0.03
25–29	51.26	1.33^*∗*^	1.68	1.67	1.69	2.26	2.25	2.27	51.23	0.03
30–34	49.74	1.29^*∗*^	1.58	1.57	1.59	2.52	2.50	2.54	53.79	0.03
35–39	52.09	1.35^*∗*^	1.74	1.73	1.75	2.88	2.86	2.90	56.86	0.05
40–44	54.78	1.42^*∗*^	1.94	1.92	1.96	3.20	3.17	3.23	59.28	0.09
≥45	52.05	1.35^*∗*^	1.74	1.69	1.78	3.07	2.98	3.16	58.32	0.33

*Parity*										
0	43.75	1.00	1.00	Reference		1.00	Reference		47.33	0.03
1	50.10	1.15^*∗*^	1.29	1.29	1.29	1.14	1.14	1.15	50.46	0.03
2	55.18	1.26^*∗*^	1.58	1.58	1.59	1.19	1.19	1.20	51.53	0.04
3	59.63	1.36^*∗*^	1.90	1.89	1.91	1.28	1.27	1.28	53.12	0.06
≥4	63.14	1.44^*∗*^	2.20	2.19	2.22	1.33	1.32	1.34	54.06	0.07

*Marital status*										
Married	47.63	1.00	1.00	Reference		1.00	Reference		49.61	0.02
Unmarried	52.92	1.11^*∗*^	1.24	1.23	1.24	1.02	1.01	1.02	50.00	0.03

*Nativity/immigrant status*										
US-born	51.00	1.12^*∗*^	1.25	1.25	1.26	1.44	1.43	1.45	51.57	0.02
Foreign-born	45.39	1.00	1.00	Reference		1.00	Reference		43.06	0.04

*Maternal education (years)*										
<12	52.95	1.33^*∗*^	1.71	1.70	1.72	1.75	1.74	1.76	53.58	0.04
12	53.83	1.36^*∗*^	1.77	1.76	1.78	1.78	1.77	1.79	53.98	0.03
13–15	54.88	1.38^*∗*^	1.85	1.84	1.85	1.76	1.75	1.76	53.69	0.03
≥16	39.70	1.00	1.00	Reference		1.00	Reference		40.37	0.03

*Place of residence*										
Metropolitan county	48.72	1.00	1.00	Reference		1.00	Reference		48.69	0.02
Nonmetropolitan county	53.54	1.10^*∗*^	1.21	1.21	1.22	1.23	1.23	1.23	53.56	0.03

*Region of residence*										
New England	45.21	0.97^*∗*^	0.94	0.93	0.95	1.16	1.15	1.17	49.54	0.09
Mid-Atlantic	46.65	1.00	1.00	0.99	1.00	1.13	1.13	1.14	49.01	0.05
East Northcentral	52.05	1.11^*∗*^	1.24	1.23	1.24	1.31	1.31	1.32	52.50	0.04
West Northcentral	50.96	1.09^*∗*^	1.18	1.18	1.19	1.26	1.25	1.26	51.43	0.06
South Atlantic	50.34	1.08^*∗*^	1.16	1.15	1.16	1.16	1.15	1.17	49.57	0.04
East Southcentral	51.99	1.11^*∗*^	1.23	1.23	1.24	1.22	1.21	1.23	50.70	0.07
West Southcentral	50.78	1.09^*∗*^	1.18	1.17	1.18	1.11	1.10	1.11	48.44	0.04
Mountain	46.74	1.00	1.00	Reference		1.00	Reference		46.09	0.06
Pacific	48.86	1.05^*∗*^	1.09	1.08	1.09	1.16	1.16	1.17	49.60	0.04

OR = odds ratio; CI = confidence interval. ^*∗*^Statistically significant at *p* < 0.05. ^1^Unadjusted for the effects of other covariates. ^2^Adjusted for race/ethnicity, maternal age, parity, marital status, nativity, maternal education, and place and region of residence. Source: data derived from the 2012–2014 US National Natality data files.

**Table 3:** Racial/ethnic variation in selected sociodemographic risk factors for pre-pregnancy overweight or obesity among women, United States, 2012–2014 (*N*=10, 431, 092).

Race/ethnicity	Maternal age <20 years	Maternal age ≥35 years	Parity ≥4	Maternal education <12 years	Maternal education ≥16 years	Unmarried mother	Foreign-born	Western or Pacific region
	Percent	Percent	Percent	Percent	Percent	Percent	Percent	Percent
All races	7.1	15.2	11.9	15.9	30.5	40.3	21.9	24.8
Non-Hispanic white	5.1	15.4	9.8	8.4	38.8	29.34	6.15	20.18
Non-Hispanic black	10.8	11.8	15.8	17.7	15.0	71.40	14.21	8.67
American Indian/AN	12.3	9.2	20.4	22.5	9.3	66.26	1.37	43.92
Chinese	0.3	31.1	2.0	9.1	66.1	11.21	87.29	47.07
Japanese	0.4	48.5	3.6	1.1	66.9	6.95	76.15	56.11
Filipino	1.5	31.5	7.0	2.3	53.8	20.09	73.75	62.59
Asian Indian	0.5	17.8	2.9	4.8	77.0	3.50	90.09	28.14
Korean	0.4	34.9	3.3	0.8	75.3	7.24	80.97	43.67
Vietnamese	1.2	30.2	5.7	11.9	38.7	19.94	83.72	45.94
Hawaiian	6.4	13.9	19.0	12.8	18.0	46.94	5.07	55.76
Samoan	6.3	12.8	26.8	11.9	7.5	39.45	32.38	86.24
Other API	4.1	17.8	13.3	16.8	34.3	28.79	62.52	41.11
Mexican	11.2	13.8	17.7	37.8	8.4	51.49	51.13	47.89
Puerto Rican	11.7	11.4	12.8	21.7	14.6	63.42	18.26	7.72
Cuban	4.7	17.5	5.4	9.0	25.1	50.21	52.56	8.43
Central/South American	5.9	20.2	13.6	38.6	17.4	49.65	82.75	22.90
Other Hispanic	12.5	11.4	13.0	24.8	12.9	55.96	26.81	41.06

Source: data derived from the 2012–2014 US National Natality data files. AN = Alaska Native.

**Table 4:** Observed prevalence and adjusted odds and prevalence of pre-pregnancy obesity (BMI ≥30) among women in 31 ethnic-immigrant groups, United States, 2012–2014 (*N*=10, 431, 092).

Ethnic-immigrant group	Number of births	Prevalence percent	Prevalence ratio	Model 1^1^			Model 2^2^			Covariate-adjusted	
				OR	95% CI		OR	95% CI		Prevalence	SE
Non-Hispanic white, US-born	53,65,062	22.94	1.00	1.00	Reference		1.00	Reference		23.16	0.02
Non-Hispanic white, immigrant	3,51,397	12.74	0.56^*∗*^	0.49	0.49	0.50	0.51	0.51	0.52	13.56	0.06
Non-Hispanic black, US-born	12,97,869	35.68	1.56^*∗*^	1.86	1.86	1.87	1.83	1.82	1.84	35.10	0.05
Non-Hispanic black, immigrant	2,14,957	23.61	1.03^*∗*^	1.04	1.03	1.05	0.94	0.93	0.95	22.14	0.09
American Indian/Alaska Native	96,037	34.86	1.52^*∗*^	1.80	1.77	1.82	1.63	1.61	1.66	32.67	0.15
Chinese, US-born	18,330	7.09	0.31^*∗*^	0.26	0.24	0.27	0.34	0.32	0.36	9.45	0.24
Chinese, immigrant	1,25,839	1.92	0.08^*∗*^	0.07	0.06	0.07	0.07	0.07	0.08	2.25	0.05
Japanese, US-born	4,305	10.76	0.47^*∗*^	0.41	0.37	0.45	0.49	0.44	0.54	12.99	0.55
Japanese, immigrant	13,746	2.22	0.10^*∗*^	0.08	0.07	0.09	0.08	0.07	0.09	2.43	0.14
Hawaiian	2,041	32.44	1.41^*∗*^	1.61	1.47	1.77	1.53	1.39	1.68	31.26	1.00
Filipino, US-born	21,876	17.51	0.76^*∗*^	0.71	0.69	0.74	0.81	0.78	0.84	19.64	0.28
Filipino, immigrant	61,472	8.61	0.38^*∗*^	0.32	0.31	0.33	0.33	0.32	0.34	9.23	0.12
Asian Indian, US-born	16,211	11.59	0.51^*∗*^	0.44	0.42	0.46	0.58	0.55	0.60	14.93	0.31
Asian Indian, immigrant	1,47,308	8.99	0.39^*∗*^	0.33	0.33	0.34	0.43	0.42	0.43	11.57	0.09
Korean, US-born	7,800	6.83	0.30^*∗*^	0.25	0.23	0.27	0.32	0.29	0.34	8.85	0.36
Korean, immigrant	33,191	4.27	0.19^*∗*^	0.15	0.14	0.16	0.18	0.17	0.19	5.19	0.13
Vietnamese, US-born	9,682	9.22	0.40^*∗*^	0.34	0.32	0.37	0.40	0.38	0.43	10.99	0.34
Vietnamese, immigrant	49,791	2.10	0.09^*∗*^	0.07	0.07	0.08	0.07	0.06	0.07	1.99	0.06
Samoan	5,336	60.23	2.63^*∗*^	5.09	4.82	5.37	4.95	4.68	5.23	58.51	0.66
Other API, US-born	57,723	21.18	0.92^*∗*^	0.90	0.88	0.92	0.97	0.95	0.99	22.64	0.18
Other API, immigrant	96,281	12.31	0.54^*∗*^	0.47	0.46	0.48	0.45	0.44	0.46	12.11	0.10
Mexican, US-born	7,28,465	31.16	1.36^*∗*^	1.52	1.51	1.53	1.61	1.60	1.62	32.33	0.06
Mexican, immigrant	7,62,159	24.88	1.08^*∗*^	1.11	1.11	1.12	0.92	0.92	0.93	21.77	0.05
Puerto Rican, mainland US-born	1,30,522	29.13	1.27^*∗*^	1.38	1.36	1.40	1.40	1.38	1.42	29.47	0.13
Puerto Rican, Puerto Rico-born	29,148	26.33	1.15^*∗*^	1.20	1.17	1.23	1.21	1.18	1.24	26.60	0.26
Cuban, US-born	24,274	24.00	1.05^*∗*^	1.06	1.03	1.09	1.14	1.11	1.17	25.48	0.28
Cuban, immigrant	26,898	14.44	0.63^*∗*^	0.57	0.55	0.59	0.51	0.50	0.53	13.58	0.20
Central/South American, US-born	54,615	25.78	1.12^*∗*^	1.17	1.15	1.19	1.32	1.30	1.35	28.33	0.20
Central/South American, immigrant	2,61,909	18.70	0.81^*∗*^	0.77	0.77	0.78	0.67	0.66	0.68	16.94	0.07
Other Hispanic, US-born	2,82,921	29.39	1.28^*∗*^	1.40	1.39	1.41	1.49	1.47	1.50	30.68	0.09
Other Hispanic, immigrant	1,03,647	20.62	0.90^*∗*^	0.87	0.86	0.89	0.79	0.77	0.80	19.25	0.12

OR = odds ratio; CI = confidence interval; API = Asian/Pacific Islander. ^*∗*^Statistically significant at *p* < 0.05. ^1^Unadjusted for the effects of other covariates. ^2^Adjusted for race/ethnicity, maternal age, parity, marital status, nativity, maternal education, and place and region of residence. Source: data derived from the 2012–2014 US National Natality data files.

**Table 5:** Observed prevalence and adjusted odds and prevalence of pre-pregnancy overweight or obesity (BMI ≥25) among women in 31 ethnic-immigrant groups, United States, 2012–2014 (*N*=10, 431, 092)

Ethnic-immigrant group	Prevalence percent	Prevalence ratio	Model 1^1^			Model 2^2^			Covariate-adjusted	
			OR	95% CI		OR	95% CI		Prevalence	SE
Non-Hispanic white, US-born	47.06	1.00	1.00	Reference		1.00	Reference		47.17	0.02
Non-Hispanic white, immigrant	35.87	0.76^*∗*^	0.63	0.63	0.63	0.64	0.64	0.65	36.80	0.08
Non-Hispanic black, US-born	61.70	1.31^*∗*^	1.81	1.81	1.82	1.84	1.83	1.85	61.66	0.05
Non-Hispanic black, immigrant	56.11	1.19^*∗*^	1.44	1.43	1.45	1.32	1.31	1.34	53.93	0.11
American Indian/Alaska Native	61.69	1.31^*∗*^	1.81	1.79	1.84	1.71	1.69	1.73	60.01	0.16
Chinese, US-born	24.07	0.51^*∗*^	0.36	0.35	0.37	0.43	0.42	0.45	28.39	0.34
Chinese, immigrant	12.10	0.26^*∗*^	0.16	0.15	0.16	0.17	0.16	0.17	13.42	0.10
Japanese, US-born	30.27	0.64^*∗*^	0.49	0.46	0.52	0.55	0.51	0.59	33.27	0.72
Japanese, immigrant	10.48	0.22^*∗*^	0.13	0.13	0.14	0.13	0.12	0.14	10.91	0.27
Hawaiian	61.29	1.30^*∗*^	1.78	1.63	1.95	1.72	1.57	1.88	60.15	1.07
Filipino, US-born	43.79	0.93^*∗*^	0.88	0.85	0.90	0.96	0.93	0.98	46.10	0.33
Filipino, immigrant	30.90	0.66^*∗*^	0.50	0.49	0.51	0.51	0.50	0.52	31.62	0.19
Asian Indian, US-born	33.04	0.70^*∗*^	0.56	0.54	0.57	0.67	0.65	0.70	37.88	0.38
Asian Indian, immigrant	36.24	0.77^*∗*^	0.64	0.63	0.65	0.78	0.77	0.79	41.27	0.13
Korean, US-born	23.35	0.50^*∗*^	0.34	0.33	0.36	0.41	0.39	0.43	27.15	0.52
Korean, immigrant	18.22	0.39^*∗*^	0.25	0.24	0.26	0.28	0.27	0.29	20.38	0.23
Vietnamese, US-born	28.49	0.61^*∗*^	0.45	0.43	0.47	0.51	0.49	0.54	31.89	0.48
Vietnamese, immigrant	12.18	0.26^*∗*^	0.16	0.15	0.16	0.14	0.14	0.14	11.56	0.14
Samoan	86.28	1.83^*∗*^	7.07	6.54	7.64	6.97	6.44	7.54	85.52	0.49
Other API, US-born	46.42	0.99^*∗*^	0.97	0.96	0.99	1.04	1.02	1.06	48.07	0.20
Other API, immigrant	36.72	0.78^*∗*^	0.65	0.64	0.66	0.62	0.61	0.63	36.03	0.15
Mexican, US-born	58.45	1.24^*∗*^	1.58	1.57	1.59	1.73	1.72	1.73	60.22	0.06
Mexican, immigrant	57.47	1.22^*∗*^	1.52	1.51	1.53	1.31	1.30	1.32	53.68	0.06
Puerto Rican, mainland US-born	56.01	1.19^*∗*^	1.43	1.42	1.45	1.49	1.47	1.50	56.71	0.14
Puerto Rican, Puerto Rico-born	52.84	1.12^*∗*^	1.26	1.23	1.29	1.29	1.26	1.32	53.37	0.29
Cuban, US-born	50.45	1.07^*∗*^	1.15	1.12	1.18	1.23	1.20	1.26	52.22	0.32
Cuban, immigrant	42.81	0.91^*∗*^	0.84	0.82	0.86	0.80	0.78	0.82	41.71	0.30
Central/South American, US-born	53.73	1.14^*∗*^	1.31	1.28	1.33	1.49	1.47	1.52	56.82	0.21
Central/South American, immigrant	50.34	1.07^*∗*^	1.14	1.13	1.15	1.02	1.01	1.03	47.65	0.10
Other Hispanic, US-born	56.66	1.20^*∗*^	1.47	1.46	1.48	1.61	1.60	1.62	58.61	0.09
Other Hispanic, immigrant	51.12	1.09^*∗*^	1.18	1.16	1.19	1.09	1.08	1.11	49.30	0.15

OR = odds ratio; CI = confidence interval; API = Asian/Pacific Islander. ^*∗*^Statistically significant at *p* < 0.05. ^1^Unadjusted for the effects of other covariates. ^2^Adjusted for race/ethnicity, maternal age, parity, marital status, nativity, maternal education, and place and region of residence. Source: data derived from the 2012–2014 US National Natality data files.

**Table 6:** Observed and adjusted prevalence and odds of pre-pregnancy obesity and overweight/obesity among women in 15 joint racial/ethnic and education groups, United States, 2012-2014 (*N*=10, 326, 186)

Racial/ethnic-education group	Observed prevalence^1^		Adjusted odds ratio^2^			Adjusted prevalence^2^	
	%	SE	OR	95% CI		%	SE
*Pre-pregnancy obesity (BMI ≥30)*							
Non-Hispanic white, education ≤12 years	26.15	0.03	3.92	3.87	3.98	25.74	0.04
Non-Hispanic white, education 13–15 years	27.11	0.03	3.62	3.57	3.67	24.26	0.03
Non-Hispanic white, education 16+ years	15.52	0.02	1.68	1.65	1.70	13.12	0.02
Non-Hispanic black, education ≤12 years	32.41	0.05	6.07	5.98	6.16	34.60	0.06
Non-Hispanic black, education 13–15 years	37.11	0.07	6.56	6.46	6.66	36.32	0.07
Non-Hispanic black, education 16+ years	32.04	0.10	5.03	4.95	5.11	30.60	0.10
American Indian/AN, education ≤12 years	34.43	0.20	5.92	5.79	6.06	34.07	0.20
American Indian/AN, education 13–15 years	37.60	0.27	5.84	5.68	6.00	33.75	0.26
American Indian/AN, education 16+ years	28.05	0.48	3.43	3.27	3.60	23.33	0.41
Hispanic, education ≤12 years	26.84	0.04	5.92	5.84	6.01	34.06	0.05
Hispanic, education 13–15 years	29.26	0.06	5.29	5.22	5.37	31.67	0.06
Hispanic, education 16+ years	19.49	0.08	2.85	2.80	2.90	20.24	0.08
Asian/Pacific Islander, education ≤12 years	12.30	0.09	2.37	2.33	2.42	17.52	0.11
Asian/Pacific Islander, education 13–15 years	13.00	0.09	2.26	2.22	2.31	16.85	0.12
Asian/Pacific Islander, education 16+ years	6.22	0.04	1.00	Reference		8.31	0.05

*Pre-pregnancy overweight or obesity (BMI ≥25)*							
Non-Hispanic white, education ≤12 years	49.82	0.04	2.62	2.60	2.64	50.22	0.04
Non-Hispanic white, education 13-15 years	52.43	0.04	2.55	2.52	2.57	49.52	0.04
Non-Hispanic white, education 16+ years	38.85	0.03	1.36	1.35	1.37	34.74	0.04
Non-Hispanic black, education ≤12 years	58.00	0.06	4.09	4.05	4.13	60.84	0.06
Non-Hispanic black, education 13–15 years	64.22	0.07	4.67	4.63	4.72	63.88	0.07
Non-Hispanic black, education 16+ years	63.11	0.10	4.14	4.10	4.19	61.14	0.10
American Indian/AN, education ≤12 years	60.58	0.21	4.18	4.10	4.26	61.34	0.21
American Indian/AN, education 13–15 years	65.36	0.27	4.39	4.28	4.50	62.48	0.27
American Indian/AN, education 16+ years	55.79	0.53	2.65	2.54	2.76	50.46	0.52
Hispanic, education ≤12 years	57.04	0.04	4.47	4.43	4.50	62.85	0.04
Hispanic, education 13–15 years	58.01	0.07	3.98	3.94	4.02	60.21	0.06
Hispanic, education 16+ years	46.91	0.10	2.28	2.26	2.31	46.90	0.10
Asian/Pacific Islander, education ≤12 years	32.89	0.12	1.60	1.58	1.62	38.45	0.13
Asian/Pacific Islander, education 13–15 years	34.58	0.13	1.60	1.58	1.62	38.50	0.14
Asian/Pacific Islander, education 16+ years	25.27	0.07	1.00	Reference		28.33	0.08

OR = odds ratio; SE = standard error; CI = confidence interval; AN = Alaska Native. ^1^Weighted prevalence. ^2^Adjusted prevalence was derived from fitted logistic regression models that included the joint variable of race/ethnicity and education, age, parity, marital status, nativity, and place and region of residence.

## Data Availability

The public-use US birth data files used to analyze and support the findings of this study are in the public domain and are available online at the Centers of Disease Control and Prevention's website (https://www.cdc.gov/nchs/data_access/vitalstatsonline.htm) and are cited in reference 18 of the paper.

## References

[1] National Center for Health Statistics. Health, United States (2016). *2015 with Special Feature on Racial and Ethnic Health Disparities*.

[2] Flegal K. M., Kruszon-Moran D., Carroll M. D., Fryar C. D., Ogden C. L. (2016). Trends in obesity among adults in the United States, 2005 to 2014. *JAMA*.

[3] Singh G. K., Siahpush M., Hiatt R. A., Timsina L. R. (2010). Dramatic increases in obesity and overweight prevalence and body mass index among ethnic-immigrant and social class groups in the United States, 1976-2008. *Journal of Community Health*.

[4] Singh G. K., Lin S. C. (2013). Dramatic increases in obesity and overweight prevalence among Asian subgroups in the United States, 1992-2011. *ISRN Preventive Medicine*.

[5] National Center for Health Statistics (2015). *The National Health Interview Survey, Questionnaires, Datasets, and Related Documentation: 1976-2014 Public Use Data Files*.

[6] Hamilton B. E., Martin J. A., Osterman M. J., Curtin S. C., Matthews T. J. (2015). Births: final data for 2014. *National Vital Statistics Reports*.

[7] Fisher S. C., Kim S. Y., Sharma A. J., Rochat R., Morrow B. (2013). Is obesity still increasing among pregnant women? Prepregnancy obesity trends in 20 states, 2003-2009. *Preventive Medicine*.

[8] Siega-Riz A. M. (2012). Prepregnancy obesity: determinants, consequences, and solutions. *Advances in Nutrition*.

[9] Poston L., Harthoorn L. F., van der Beek E. M. (2011). Obesity in pregnancy: implications for the mother and lifelong health of the child. A consensus statement. *Pediatric Research*.

[10] Cnattingius S., Bergström R., Lipworth L., Kramer M. S. (1998). Prepregnancy weight and the risk of adverse pregnancy outcomes. *New England Journal of Medicine*.

[11] Stang J., Huffman L. G. (2016). Position of the academy of nutrition and dietetics: obesity, reproduction, and pregnancy outcomes. *Journal of Academy of Nutrition and Dietetics*.

[12] Hinkle S. N., Sharma A. J., Kim S. Y. (2011). Prepregnancy obesity trends among low-income women, United States, 1999-2008. *Maternal and Child Health Journal*.

[13] Kim H. H., Monteiro K., Phanthavong S., Viner-Brown S. (2015). Prepregnancy obesity and adverse health conditions in Rhode Island. *Rhode Island Medical Journal*.

[14] Declercq E., MacDorman M., Cabral H., Stotland N. (2016). Prepregnancy body mass index and infant mortality in 38 U.S. states, 2012-2013. *Obstetrics & Gynecology*.

[15] Kim S. Y., Dietz P. M., England L., Morrow B., Callaghan W. M. (2007). Trends in pre-pregnancy obesity in nine States, 1993-2003. *Obesity*.

[16] Cawthon L., Reed P. (2005). *Obesity and Pregnancy*.

[17] Branum A. M., Kirmeyer S. E., Gregory E. C. W. (2016). Prepregnancy body mass index by maternal characteristics and state: data from the birth certificate. *National Vital Statistics Report*.

[18] National Center for Health Statistics (2015). *National Vital Statistics System, 2012-2014 Natality Public Use Files and User Guide*.

[19] Singh G. K., Rodriguez-Lainz A., Kogan M. D. (2013). Immigrant health inequalities in the United States: use of eight major national data systems. *Scientific World Journal*.

[20] World Health Organization. World Health Statistics (2016). *World Health Statistics 2016: Monitoring health for the SDGs*.

[21] SAS Institute (2011). *Inc. SAS/STAT User’s Guide, Version 9.3: The LOGISTIC Procedure*.

[22] Blackwell D. L., Lucas J. W., Clarke T. C. (2012). Summary health statistics for U.S. adults: national health interview survey, 2012. *Vital and Health Statistics*.

[23] Headen I. E., Davis E. M., Mujahid M. S., Abrams B. (2012). Racial-ethnic differences in pregnancy-related weight. *Advances in Nutrition*.

[24] US Census Bureau (2014). *The American Community Survey*.

[25] Singh G. K., Lin S. C. (2013). Marked ethnic, nativity, and socioeconomic disparities in disability and health insurance among US children and adults. *BioMed Research International*.

[26] Singh G. K., Siahpush M., Kogan M. D. (2010). Neighborhood socioeconomic conditions, built environments, and childhood obesity. *Health Affairs*.

[27] Pappas M. A., Alberg A. J., Ewing R. (2007). The built environment and obesity. *Epidemiologic Reviews*.

[28] Thangaratinam S., Rogozińska E., Jolly K. (2012). Interventions to reduce or prevent obesity in pregnant women: a systematic review. *Health Technology Assessment*.

[29] Rasmussen K. M., Yaktine A. L. (2009). Institute of medicine (US) and National Research Council (US) Committee to Reexamine IOM pregnancy weight guidelines. *Weight Gain During Pregnancy: Reexamining the Guidelines*.

[30] National Heart Lung (2013). *Managing Overweight and Obesity in Adults*.

[31] Buschur E., Kim C. (2012). Guidelines and interventions for obesity during pregnancy. *International Journal of Gynecology and Obstetrics*.

[32] US Department of Health and Human Services (2017). *Healthy People 2020*.

[33] Finkelstein E. A., Trogdon J. G., Cohen J. W., Dietz W. (2009). Annual medical spending attributable to obesity: payer-and service-specific estimates. *Health Affairs*.

[34] US Department of Agriculture (2017). *Pregnancy & Breastfeeding*.

[35] King J. C. (2006). Maternal obesity, metabolism, and pregnancy outcomes. *Annual Review of Nutrition*.

